# The association between child and adolescent emotional disorder and poor attendance at school: a systematic review protocol

**DOI:** 10.1186/s13643-017-0523-6

**Published:** 2017-06-28

**Authors:** Katie Finning, Darren Moore, Obioha C. Ukoumunne, Emilia Danielsson-Waters, Tamsin Ford

**Affiliations:** 1 University of Exeter Medical School, South Cloisters, St Luke’s Campus, Exeter, EX1 2LU UK; 2 University of Bristol, Bristol, UK

**Keywords:** School attendance, Absenteeism, School refusal, Emotional disorder, Anxiety, Depression, Children, Adolescents

## Abstract

**Background:**

Anxiety and depression are common in young people and are associated with a range of adverse outcomes. Research has suggested a relationship between emotional disorder and poor school attendance, and thus poor attendance may serve as a red flag for children at risk of emotional disorder. This systematic review aims to investigate the association between child and adolescent emotional disorder and poor attendance at school.

**Methods:**

We will search electronic databases from a variety of disciplines including medicine, psychology, education and social sciences, as well as sources of grey literature, to identify any quantitative studies that investigate the relationship between emotional disorder and school attendance. Emotional disorder may refer to diagnoses of mood or anxiety disorders using standardised diagnostic measures, or measures of depression, anxiety or “internalising symptoms” using a continuous scale. Definitions for school non-attendance vary, and we aim to include any relevant terminology, including attendance, non-attendance, school refusal, school phobia, absenteeism and truancy. Two independent reviewers will screen identified papers and extract data from included studies. We will assess the risk of bias of included studies using the Newcastle-Ottawa Scale. Random effects meta-analysis will be used to pool quantitative findings when studies use the same measure of association, otherwise a narrative synthesis approach will be used.

**Discussion:**

This systematic review will provide a detailed synthesis of evidence regarding the relationship between childhood emotional disorder and poor attendance at school. Understanding this relationship has the potential to assist in the development of strategies to improve the identification of and intervention for this vulnerable group.

**Systematic review registration:**

PROSPERO CRD42016052961

**Electronic supplementary material:**

The online version of this article (doi:10.1186/s13643-017-0523-6) contains supplementary material, which is available to authorized users.

## Background

### Rationale

Emotional disorders are among the most common psychiatric disorders in children and adolescents, with worldwide prevalence estimates of 7 and 3% for anxiety and depressive disorders, respectively [1]. In addition, emotional disorders are identified as leading contributors to the burden of disease in young people [2]. In the short term, emotional disorders are associated with a range of adverse consequences including educational problems, difficulties at home and in social relationships, physical health problems, smoking and substance abuse [3, 4]. In the long term, they are associated with adult psychiatric disorder, substance abuse, increased risk of suicide, increased treatment utilisation and social impairments [5, 6]. Effective interventions for these disorders exist [7], yet rates of help-seeking remain low, with estimates suggesting that approximately 80% of children fail to access effective treatment [8, 9]. There is therefore a need to explore how emotional disorders in children and adolescents could be better detected, to allow for provision of appropriate support and intervention. Since the majority of children spend a large proportion of their time at school, teachers and other educational practitioners are well-placed to identify potential emotional disorders in young people. A recent report from the UK Department for Education (DfE) highlights the important role of schools in supporting children to be resilient and mentally healthy, and promotes the use of attendance data as one way of identifying children at risk of mental health problems [10]. In the UK, education is compulsory for all children from the school term immediately following their fifth birthday, and any child absent for 10% or more of school “sessions” (morning or afternoon) is considered “persistently absent” [11].

Traditionally, research has characterised persistent non-attendance at school in two broad categories: *school refusal*, reflecting non-attendance due to emotional distress and *truancy*, reflecting non-attendance due to a lack of interest in school or antisocial behaviour [12]. In practice, however, a broad range of labels are used, including school refusal, truancy, absenteeism, persistent non-attendance and school phobia. These terms are often used interchangeably, and there is little consensus about how best to define and assess the full spectrum of persistent non-attendance. In addition, there is evidence to suggest that school refusal and truancy are not mutually exclusive, leading some researchers to argue that a more inclusive approach is needed that makes no assumptions about the underlying aetiology [13]. Emotional disorder can be conceptualised in different ways but is generally considered to mean depression or anxiety [14]. Some studies examine diagnoses using standardised diagnostic measures, and others measure symptoms of depression or anxiety on a continuous scale. Studies may also refer to “internalising problems” or “internalising symptoms”, which are generally considered to mean a combination of depression and anxiety [15].

Studies have shown a relationship between emotional disorder and non-attendance at school. For example, McShane et al [16] found that 54 and 52% of children assessed or treated for school refusal in an Australian clinic had diagnoses of anxiety and mood disorders, respectively, and a large USA community sample found that 25% of anxious school refusers and 88% of mixed school refusers (i.e. those meeting criteria for both anxious school refusal and truancy) met criteria for a psychiatric disorder, compared to 7% of non-school refusers [17]. Somatic symptoms such as headaches, stomach aches and fatigue are commonly reported in children with emotional disorders, and there is some evidence that somatisation may be one of the pathways through which emotional disorders impact on school attendance [18]. Research investigating the relationship between emotional disorder and poor attendance at school has varied extensively in setting, sample sizes, use of comparators and definitions and methods of assessing both attendance and emotional disorder, making comparison between studies difficult, and we are aware of no systematic reviews that have addressed this relationship. Understanding the association between emotional disorder and poor attendance at school has the potential to assist in the development of strategies to improve the identification of and intervention for children with emotional disorder.

### Objectives

The objectives of this systematic review are to address the following research questions:

- Is there an association between child and adolescent emotional disorder and poor attendance at school?
- Is this association moderated by between-study characteristics such as age of child, type of emotional disorder, somatic symptoms, measurement source (e.g. child-report, parent-report), assessment method (diagnostic tool or measures of continuous symptoms), study setting or type of school?

## Methods

This protocol follows the Preferred Reporting Items for Systematic review and Meta-Analysis Protocols (PRISMA-P) guidelines [19] (see Additional file 1) and is registered on PROSPERO, an international register of systematic reviews [20] (CRD42016052961). Any changes to the protocol will be recorded on PROSPERO.

### Eligibility criteria

#### Population

Participants will be school-aged children and adolescents. Although research into school attendance typically focuses on children aged 5–17 [13], we expect the exact age range to vary between studies, and we will include any age ranges applicable for the country of study. We will exclude retrospective reports collected during adulthood, and studies where the sample is a specific population not comparable to other study samples, for example, those focusing on children with a particular health condition.

#### Main study variables

We intend to include any study that investigates the relationship between emotional disorder and school attendance. Based on a previous systematic review on a similar topic [21] and an initial scoping search, we anticipate that studies will vary in the way they investigate the relationship between these variables, with some treating emotional disorder as the exposure and school attendance as the outcome and others considering poor attendance the exposure and emotional disorder the outcome. Emotional disorder must be assessed by diagnosis with a standardised diagnostic measure or by measures of symptoms using a validated scale. This will allow for inclusion of studies reporting on children with a diagnosed emotional disorder, as well as those with higher levels of emotional symptoms compared to their peers. We anticipate that a broad range of methods will be applied to assess and define school attendance and will take an inclusive approach. Methods of assessing attendance can include, but will not be limited to: a quantitative measure of actual attendance, or assessment of attendance behaviours using structured interviews or self-report measures. We will include any terminology used for attendance including, but not restricted to: attendance, non-attendance, school refusal, school phobia, absenteeism and truancy.

#### Types of studies

Quantitative studies of any design that report on the relationship between emotional disorder and school attendance will be eligible, including population surveys and case-control, cross-sectional, longitudinal and cohort studies. Studies where the primary aim was to evaluate the effectiveness of an intervention will be excluded, as will case studies, case series, editorials or opinion pieces and papers published in languages other than English. No restrictions will be placed on country of study. Publications that do not provide enough information to allow for quality appraisal, for example, conference abstracts, will be excluded.

### Information sources

Studies will be identified from the following information sources:

1. Electronic database searching: we will search electronic databases from date of inception to present, as we do not have reason to believe the concepts of emotional disorder or school attendance are likely to have changed substantially over time. The following databases will be searched using a pre-specified search strategy (see ‘search strategy’):
  MEDLINE (via OvidSP), including Epub Ahead of Print, In-Process & Other Non-Indexed Citations, Ovid MEDLINE Daily and Ovid MEDLINE
  PsycINFO (via OvidSP)
  Education Resources Information Centre (ERIC) (via EBSCOhost)
  Applied Social Sciences Index and Abstracts (ASSIA) (via ProQuest)
  Education Research Complete (via EBSCOhost)
  British Education Index (via EBSCOhost)
  Australian Education Index (via ProQuest)
  ProQuest Dissertations and Theses (PQDT) (via ProQuest)
2. Grey literature: grey literature is usually understood to mean literature that is not formally published in sources such as books or journal articles [22], and its inclusion in a systematic review minimises the impact of publication bias. We will search Health Management Information Consortium (HMIC), Conference Proceedings Citation Index, ProQuest Dissertations and Theses and the website OpenGrey (via http://www.opengrey.eu/), all of which index grey literature.
3. Citation searching: citation lists of included studies will be checked (backward citation searching), as will papers that have cited included studies, according to Web of Science (forward citation searching).
4. Experts in the field: experts in the field and corresponding authors of included papers will be contacted for any further information, such as unpublished material or papers in press.

### Search strategy

A comprehensive search strategy has been developed following a scoping review of the topic area and consultation with an information specialist as well as experts in the fields of child mental health and education. The search strategy will include both free-text searching and controlled vocabulary searching (e.g. MEDLINE Medical Subject Headings (MeSH) terms). Terms will be grouped according to four concepts:

- Child terms (e.g. child, adolescent, student, pupil)
- Setting terms (e.g. school, education, nursery)
- Attendance terms (e.g. attendance, non-attendance, refusal)
- Emotional disorder terms (e.g. emotional disorder, anxiety, depression, internalizing)

The master search strategy for MEDLINE (OvidSP) can be found in Additional file 2.

### Study records

#### Selection process

An initial screening of titles and abstracts of identified papers will be performed by two independent reviewers, who will assess each study for relevance according to the pre-specified eligibility criteria. Studies that cannot be conclusively excluded from title and abstract screening will be taken forward to full text screening, at which stage the full text will be obtained and a second screening process performed, again by two independent reviewers. This will result in a final set of papers to be included in the review. Discrepancies between the two reviewers at any stage will be resolved through discussion and, if required, referral to a third reviewer. The number of studies identified, included and excluded at each stage will be reported using a PRISMA flow diagram [23] together with reasons for exclusion at the full-text stage.

#### Data management

EndNote X7 software will be used to manage references throughout the review process. The results of searches will be exported to EndNote and duplicates will be removed.

#### Data extraction

A data extraction form will be developed specifically for the current study, guided by the full-text screening stage and following templates provided by experts in systematic reviewing. The data extraction form will be pilot tested on three studies selected from those to be included in the review. Data extraction will be completed by two independent reviewers for all included studies. Where there are multiple publications from one study, we will treat all sources as one and extract the data into one form. Data items to be extracted will include: study design, age and gender of participants, country of study, sample size, setting, method of assessing emotional disorder, measures of school (non-)attendance and relevant findings including effect sizes, confidence intervals and *p* values, where provided.

### Risk of bias in individual studies

Assessing risk of bias in included studies is important in determining the validity of the results and interpretation of findings. Risk of bias of included studies will be assessed by two independent reviewers using the Newcastle-Ottawa scale (NOS), which is a widely used scale designed for use in epidemiological systematic reviews [24]. The risk of bias for each included study will be taken into consideration during data synthesis.

### Data synthesis

Study characteristics will be summarised using means and standard deviations (or medians and interquartile ranges) for continuous variables, and numbers and percentages for categorical variables. A narrative report of study characteristics will also be provided. Where at least two studies have used the same method to analyse the relationship between emotional disorder and school attendance, random effects meta-analysis (using the DerSimonian and Laird method [25]) will be used to pool the quantitative results across studies. The pooled “effect” estimate (e.g. mean difference, standardised mean difference, risk ratio etc) will be reported with 95% confidence interval and *p* value. Heterogeneity will be quantified using the I-squared statistic, and we will assess the presence of heterogeneity using Cochran’s Q test. If there are sufficient data, we will perform subgroup analyses to investigate the effects of the following potential moderator variables on the relationship between emotional disorder and poor school attendance: mean age of children, type of emotional disorder, somatic symptoms, measurement source (e.g. child-report, parent-report), assessment method (diagnostic tool or measures of continuous symptoms), study setting and type of school. If subgroup analyses show statistically significant evidence that two or more of these variables are moderators, and there are at least ten studies per moderator, then we will consider using meta-regression to examine the moderators in a single model. If there are sufficient studies, we will also examine the possibility of publication bias using funnel plots and Egger’s regression test [26]. We will use the assessment of risk of bias in individual studies to indicate the strength of the body of evidence for each main outcome variable.

## Discussion

This systematic review will provide a detailed synthesis of the evidence base regarding the relationship between emotional disorder in children and adolescents and poor attendance at school. There are a number of studies that have reported on this relationship, but due to the heterogeneity in settings, definitions and methods of assessment, comparison between studies is difficult. Our synthesis will take into account the strengths and limitations of the included studies, as well as any limitations in our own methodology, and we will consider factors that may explain any observed differences between studies. This is the first systematic review of which we are aware to investigate the relationship between child and adolescent emotional disorder and poor attendance at school. Understanding this relationship has the potential to assist in the development of strategies to improve the identification and intervention of this vulnerable group.

## Additional files

Additional file 1: PRISMA-P checklist. (DOCX 29 kb)

Additional file 2: Search strategy for MEDLINE. (DOCX 13 kb)

## Abbreviations

ASSIA: Applied Social Sciences Index and Abstracts
DfE: Department for Education
ERIC: Education Resources Information Centre
HMIC: Health Management Information Consortium
MeSH: Medical Subject Headings
NOS: Newcastle-Ottawa scale
PQDT: ProQuest Dissertations and Theses
PRISMA: Preferred Reporting Items for Systematic reviews and Meta-Analyses
PRISMA-P: Preferred Reporting Items for Systematic review and Meta-Analysis Protocols.
