# Supplementary material for: The association between child and adolescent emotional disorder and poor attendance at school: a systematic review protocol
Source: Syst Rev. 2017 Jun 28;6:121. doi: 10.1186/s13643-017-0523-6 (PMC5490167; doi:10.1186/s13643-017-0523-6)
Supplement: Supplementary file 2 — Search strategy for MEDLINE. (DOCX 13 kb) [file 13643_2017_523_MOESM2_ESM.docx]

Database: Epub Ahead of Print, In-Process & Other Non-Indexed Citations, Ovid MEDLINE(R) Daily and Ovid MEDLINE(R) <1946 to Present>

Search Strategy:

--------------------------------------------------------------------------------

1 child*.tw. (1244742)

2 exp child/ (1803801)

3 adolescen*.tw. (238410)

4 exp Adolescent/ (1893745)

5 student*.tw. (231324)

6 exp Students/ (104268)

7 youth*.tw. (62590)

8 pupil*.tw. (25981)

9 schoolchild*.tw. (12735)

10 (young adj (people or person)).tw. (22633)

11 1 or 2 or 3 or 4 or 5 or 6 or 7 or 8 or 9 or 10 (3434888)

12 ((school* or kindergarten or nursery or education*) adj4 (attend* or non-attend* or refus* or absen* or school phobi* or truan*)).tw. (14958)

13 11 and 12 (12367)

14 (emotional adj (disorder* or distress or symptom*)).tw. (8983)

15 depressi*.tw. (335350)

16 anxi*.tw. (171048)

17 exp anxiety disorder/ (76077)

18 affect* disorder*.tw. (15899)

19 mood disorder*.tw. (14943)

20 exp mood disorder/ (114933)

21 dysthymi*.tw. (3172)

22 bipolar.tw. (57190)

23 agoraphobi*.tw. (3328)

24 panic disorder*.tw. (9212)

25 internalising.tw. (449)

26 internalizing.tw. (7770)

27 14 or 15 or 16 or 17 or 18 or 19 or 20 or 21 or 22 or 23 or 24 or 25 or 26 (556214)

28 13 and 27 (1313)

29 limit 28 to english language (1153)

***************************
